# Medical Society of the Upper Illinois

**Published:** 1850-09

**Authors:** 


### ARTICLE XIII.

### MEDICAL SOCIETY OF THE UPPER ILLINOIS.

Pursuant to notice, a meeting of physicians was held in Lacon, Marshall, Co., July 2d, 1850, for the purpose of estalishing a local Medical Society, auxiliary to the State Medical Society, and designed to comprise the physicians of Marshall, Bureau, Stark, Putnam, and the adjoining counties. The Convention, after organizing in due form, adopted a Constitution and By-Laws, and resolved itself into the

"Medical Society of the Upper Illinois," and elected the following-named gentlemen as officers for the ensuing year:—

Dr. ALBERT REYNOLDS, of Magnolia President.
" R. Boal, of Lacon, Vice-President.
" S. Allen Paddock, of Princeton, Secretary.
" U. P. Golliday, of Lacon, Treasurer.

| | |
|---|---|
| Dr. Thomas Hall, of Toulon,<br>" Wm. O Chamberlain, Princeton,<br>" L. G. Thompson, of Lacon, | *Censors.* |

